# Supplementary material for: 4-OI Protects MIN6 Cells from Oxidative Stress Injury by Reducing LDHA-Mediated ROS Generation
Source: Biomolecules. 2022 Sep 4;12(9):1236. doi: 10.3390/biom12091236 (PMC9496514; doi:10.3390/biom12091236)
Supplement: Supplementary file 1 [file biomolecules-12-01236-s001.zip › biomolecules-1864711-supplementary.pdf]

Supplementary Information

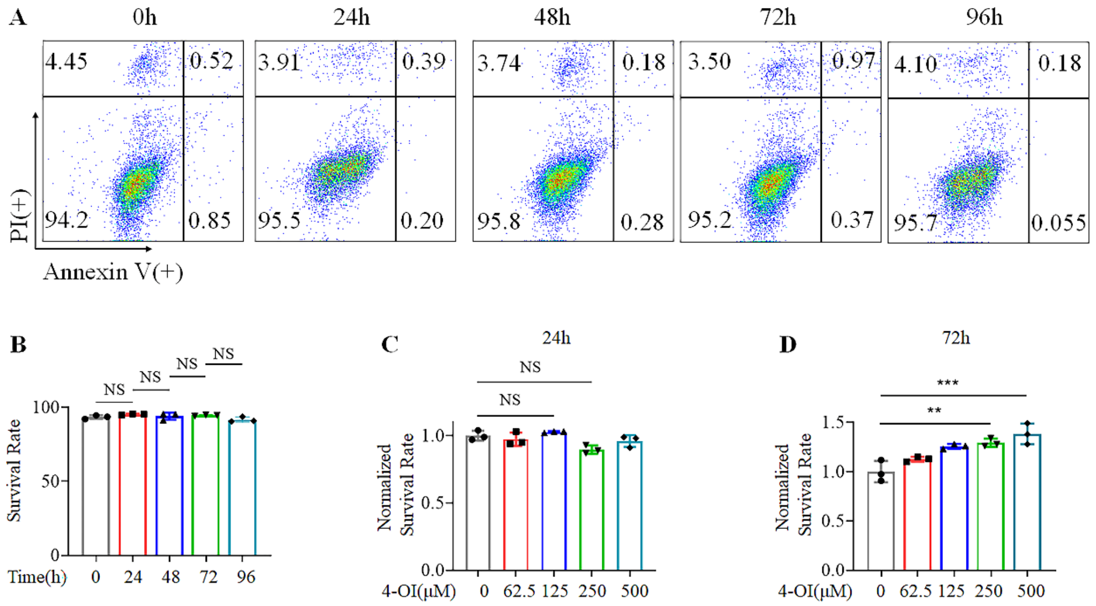

Supplementary Figure S1. The viability of MIN6 cell under various conditions. FACS analysis showing MIN6 cell viability (stained with PI and Annexin V) under normoxia at various time points (A) and its quantification (B). MTS analysis shows MIN6 cell survival rate under various 4-OI concentrations for 24 h(C) and 72 h(D) respectively. Error bars represent the mean ± SD. One-way ANOVA was performed with*p < .05, **p < .005, ***p < .0005, ****p < .00005, ns: non-significant.

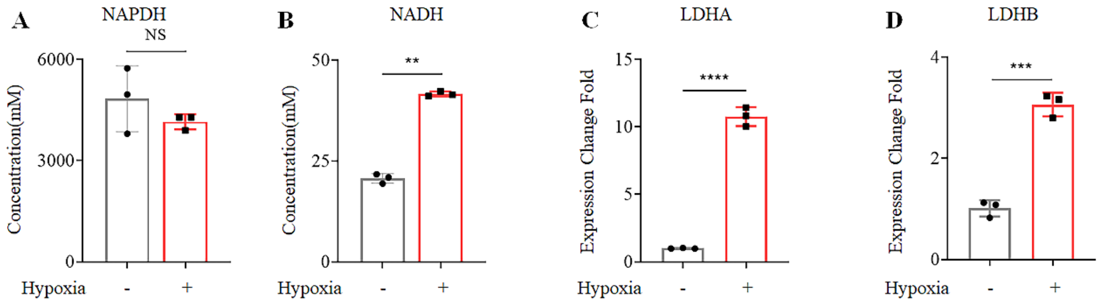

Supplementary Figure S2. The NAPDH, NADH, LDHA and LDHB levels in cells cultured under hypoxia. (A, B) Cellular NADPH and NADH levels under hypoxia for 72h measured by GPX activity assay kit and GAPDH activity assay kit respectively. (C, D) The gene expression of LDHA and LDHB was assessed by Quantitative Real-Time PCR in cells cultured under hypoxia for 72 h. Error bars represent the mean ± SD. An unpaired t-test was performed with*p < .05, **p < .005, ***p < .0005, ****p < .00005, ns: non-significant.

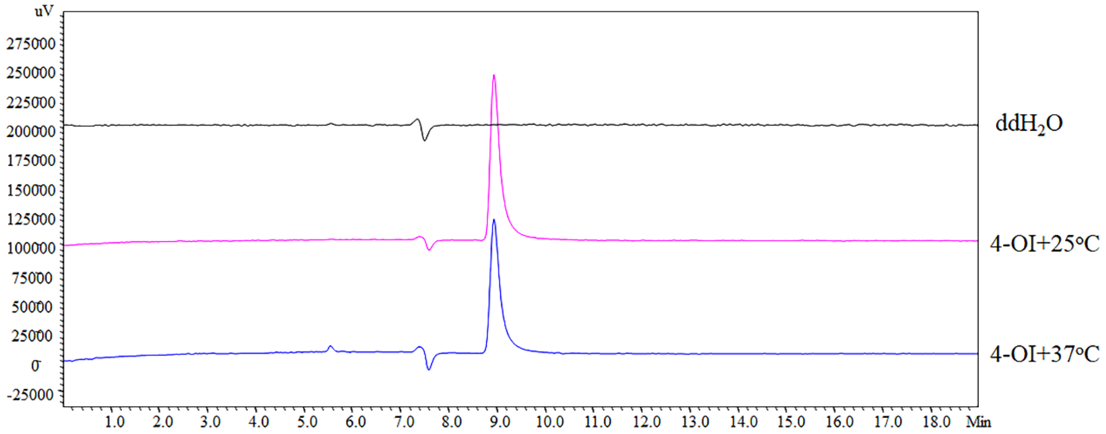

Supplementary Figure S3. HPLC was performed to verify that incubation of 4-OI at 37°C would decompose and release itaconate.

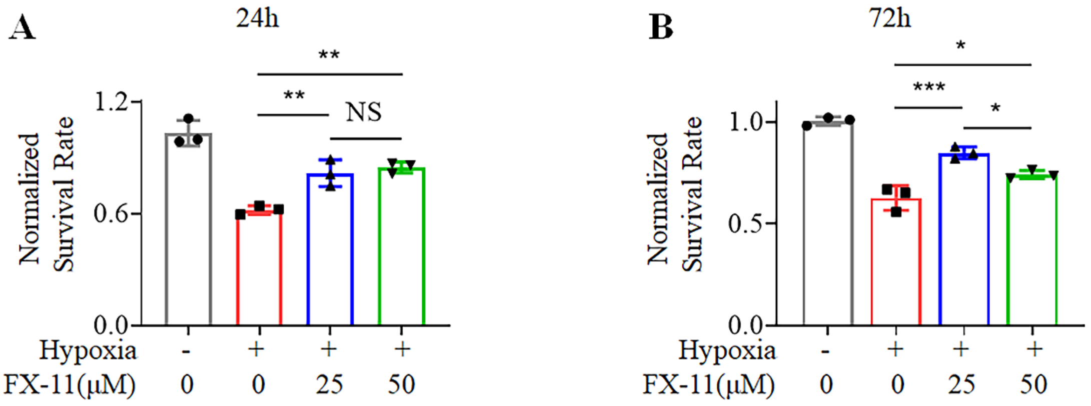

Supplementary Figure S4. The survival rate of MIN6 β-cells was shown by MTS assays when treated with different concentrations of FX-11 for 24h(A) or 72h(B) under hypoxia. Error bars represent the mean ± SD. One-way ANOVA was performed with*p < .05, **p < .005, ***p < .0005, ****p < .00005, ns: non-significant.

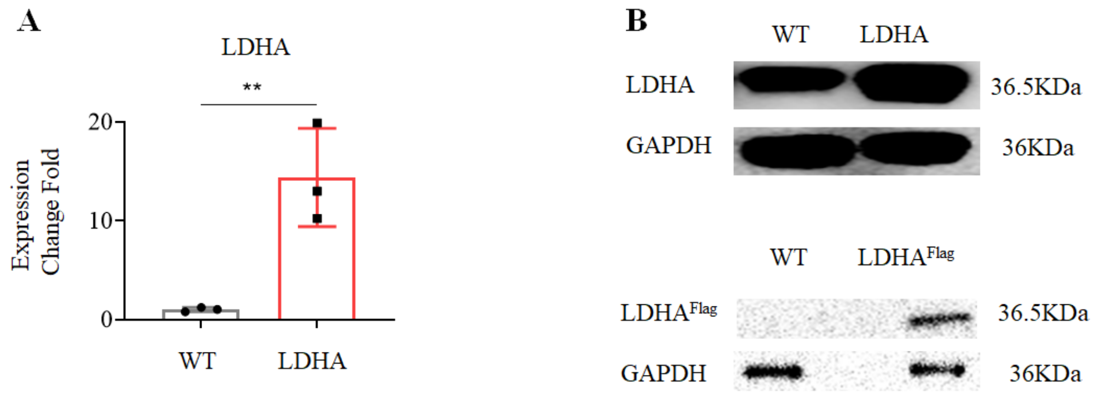

Supplementary Figure S5. Over expression of LDHA in MIN6 β-cells. (A)The level of LDHA after transfection in cells was assessed by qPCR. (B)The expression of LDHA and LDHAFlag in transfected MIN6 β-cells was verified by Western blot. Error bars represent the mean ± SD. An unpaired t-test was performed with*p < .05, **p < .005, ***p < .0005, ****p < .00005, ns: non-significant.

Supplementary Table S1. qPCR primer list

Gene Name	Primer Sequence
TNF	F- GGTGCCTATGTCTCAGCCTCTT
	R- GCCATAGAACTGATGAGAGGGAG
IL-1	F-TGGACCTTCCAGGATGAGGACA
	R-GTTCATCTCGGAGCCTGTAGTG
IL-6	F- ACGCAGACAAGGAGCAGTGGAA
	R- ATGCTCTCAGCCAAGTCTGCCA
LDHA (For overexpression)	F-CCCAAGCTTATGGCAACTCTAAAGGATCAG
	R- CCGGAATTCTTAAAATTGCAGCTCCTTTTG
LDHA (For qPCR)	F-ACGCAGACAAGGAGCAGTGGAA
	R-ATGCTCTCAGCCAAGTCTGCCA
LDHB	F- CCTCAGATCGTCAAGTACAGCC
	R-ATCCGCTTCCAATCACACGGTG

Supplementary Table S2. Antibody of Western blot list

Antibody Name	Catalog Number	Source	Molecular weight	Company
Cleaved Caspase3	9661s	Rabbit	17,19KDa	CST, USA
LC3B	3868T	Rabbit	14,16KDa	CST, USA
HIF1-α	A17906	Rabbit	120 KDa	ABclonal, China
MLKL	66675-1-Ig	Mouse	54KDa	Proteintech, USA
RIPK3	17563-1-AP	Rabbit	57KDa	Proteintech, USA
GAPDH	60004-1-Ig	Mouse	36KDa	Proteintech, USA
β-actin	66009-1-Ig	Mouse	42KDa	Proteintech, USA
